# High Prevalence of Cryptococcal Antigenemia among HIV-infected Patients Receiving Antiretroviral Therapy in Ethiopia

**DOI:** 10.1371/journal.pone.0058377

**Published:** 2013-03-04

**Authors:** Abere Shiferaw Alemu, Russell R. Kempker, Admasu Tenna, Christopher Smitson, Nega Berhe, Daniel Fekade, Henry M. Blumberg, Abraham Aseffa

**Affiliations:** 1 Haramya University, Medical Laboratory Technology, Harar, Ethiopia; 2 Division of Infectious Diseases Department of Medicine, Emory University School of Medicine, Atlanta, Georgia, United States of America; 3 Division of Infectious Diseases, Department of Medicine, Addis Ababa University, Addis Ababa, Ethiopia; 4 Aklilu Lemma Institute of Pathobiology, Addis Ababa University, Addis Ababa, Ethiopia; 5 Departments of Epidemiology and Global Health, Rollins School of Public Health of Emory University, Atlanta, Georgia, United States of America; 6 Armauer Hansen Research Institute, Addis Ababa, Ethiopia; Fundacion Huesped, Argentina

## Abstract

**Background:**

Cryptococcal disease is estimated to be responsible for significant mortality in Sub-Saharan Africa; however, only scarce epidemiology data exists. We sought to evaluate the prevalence of and risk factors for cryptococcal antigenemia in Ethiopia.

**Methods:**

Consecutive adult HIV-infected patients from two public HIV clinics in Addis Ababa, Ethiopia were enrolled into the study. A CD4 count ≤200 cells/μl was required for study participation. Patients receiving anti-retroviral therapy (ART) were not excluded. A cryptococcal antigen test was performed for all patients along with an interview, physical exam, and medical chart abstraction. Logistic regression analysis was used to assess risk factors for cryptococcal antigenemia.

**Results:**

369 HIV-infected patients were enrolled; mean CD4 123 cells/μl and 74% receiving ART. The overall prevalence of cryptococcal antigenemia was 8.4%; 11% in patients with a CD4 count <100 cells/μl, 8.9% with CD4 100 to 150 cells/μl and 5.7% with CD4150-200 cell/μl. 84% of patients with cryptococcal antigenemia were receiving ART. In multivariable analysis, increasing age, self reported fever, CD4 count <100 cells/μl, and site of screening were associated with an increased risk of cryptococcal antigenemia. No individual or combination of clinical symptoms had optimal sensitivity or specificity for cryptococcal antigenemia.

**Conclusion:**

Cryptococcal antigenemia is high in Ethiopia and rapid scale up of screening programs is needed. Screening should be implemented for HIV-infected patients with low CD4 counts regardless of symptoms or receipt of ART. Further study into the effect of location and environment on cryptococcal disease is warranted.

## Introduction

Increasing access to antiretroviral therapy (ART) has transformed the prognosis of HIV-infected patients in resource-limited settings. However, treatment coverage remains relatively low, and HIV diagnosis occurs at a late stage [1]. The high burden of opportunistic infections remains an enormous challenge to optimal HIV care and in resource-limited settings (RLS), patients continue to die of HIV-related opportunistic infections (OIs) in the weeks prior to, and months following initiation of ART. In particular, recent reports highlight the alarming issue of cryptococcal meningitis (CM) in Sub-Saharan Africa (SSA) and make it clear that there is still much to be done to improve the diagnosis and management of CM [2], [3]. Although data is limited on the prevalence of CM in much of SSA, it is estimated there are >700,000 cases of CM in SSA annually resulting in >500,000 deaths [2]. The high case fatality rate is due in large part to the lack of diagnostics and appropriate treatment options in RLS. The tragic situation of CM in SSA and other resource-limited settings presents a tremendous opportunity for various stakeholders to work together to confront the emerging CM epidemic.

The World Health Organization (WHO) has recently released “rapid advice” guidelines for cryptococcal disease among persons living with HIV which are focused on RLS [1]. Early diagnosis is key to reducing mortality due to cryptococcal disease. A major WHO recommendation is to consider implementation of cryptococcal antigen screening and pre-emptive anti-fungal therapy in those with a positive diagnostic test among ART-naïve adults with a CD4 count <100 cell/μl^3^ in areas with a high prevalence of cryptococcal disease.^3^ The recommendation is supported by epidemiological and clinical studies demonstrating a high prevalence of cryptococcal antigenemia among ART-naïve adults in several RLS, [4]–[7] increased one-year mortality in patients with cryptococcal antigenemia, [4], [5] and the cost effectiveness of screening and treatment of HIV-infected patients with cryptococcal antigenemia [8]. Additionally, data demonstrating cryptococcal antigenemia may precede the development of CM by up to 22 days add to the scientific rationale of a screen and treat strategy [9].

One limitation to implementing the WHO guidelines is that the prevalence of cryptococcal infection is not known in many countries in SSA, related in large part to lack of diagnostic capacity for cryptococcus and other HIV-related OIs. The purpose of our study was to determine the prevalence of and risk factors for cryptococcal antigenemia among HIV-infected adults attending two large public HIV clinics in Addis Ababa, Ethiopia. Currently no data exists on the extent of cryptococcal infection in Ethiopia, the second largest country in Africa with an estimated 1.1 million persons living with HIV [10]. An improved understanding of the epidemiology of cryptococcal infection is important in designing, studying, and implementing effective cryptococcal intervention strategies in Ethiopia and other similar countries in SSA.

## Methods

### Study Design and Patients

We performed a cross sectional study among HIV-infected patients in Addis Ababa, Ethiopia attending two large public HIV clinics. Consecutive patients were enrolled between May and August 2011 from the outpatient ART clinics of both Tikur Anbessa (Black Lion) Hospital and ALERT hospital, which have over 1,000 and 6,000 registered HIV-infected patients, respectively. Patients ≥18 years old and with a CD4 count ≤200 cells/μl were enrolled during a routine clinic visit. Study participants were not required to be ART naïve. Patients treated for cryptococcal infection in the last three months or currently taking an antifungal agent were excluded from study participation.

### Ethics Statement

The study was conducted according to the principles of the Declaration of Helsinki. Written informed consent was required from all study participants and the study was approved by the Institutional Review Boards of Emory University, Addis Ababa University, and Armauer Hansen Research Institute (AHRI)/ALERT Hospital. All samples were de-identified of personal identifiers for data entry and data analysis.

### Procedures

Patient interview, a physical exam, and medical chart review and abstraction were performed for each study participant to collect information regarding demographics, medical history, and clinical signs and symptoms. Specific information from the medical chart was collected on ART use, opportunistic infection history, and the most recent CD4 count.

A blood sample was obtained from each study participant to perform cryptococcal antigen testing using the Meridian Cryptococcal Latex Agglutination System (Meridian Bioscience, Cincinnati, OH, USA), a simple FDA-approved latex agglutination test capable of detecting the capsular polysaccharide of *Cryptococcus neoformans* in blood. Serum was separated from the blood sample and then frozen at – 20°C. Serum samples were batched and cryptococcal antigen testing was performed every 5–7 days at the Armauer Hansen Research Institute (AHRI) laboratory according to the manufacturer's recommendation. In brief, 200 μl of thawed serum sample was added to 200 μl of pronase solution, incubated at 56°C for 15 minutes, and then boiled for 5 minutes. After cooling, 25 μl of the specimen was placed on an agglutination card along with one drop of detection latex and then the card was shaken to mix the contents together. Positive and negative controls were also included. Results were read out visually based on degree of agglutination and rated on a scale ranging from 0 to 4+. A reading of 2+ was considered positive. For all positive samples, specimens were further tested after serial dilutions from 1∶4 up to 1∶1024. The same laboratory technician performed all cryptococcal antigen latex agglutination testing. All test results were communicated to each patient's physician.

### Data Analysis

All data was entered into an online REDCap database [11] and analyzed using SAS version 9.3 (SAS institute, Cary, NC, USA). For comparing characteristics among patients with a positive or negative cryptococcal antigen test result, differences in categorical variables were tested using χ^2^ and for continuous variables a two-sample *t*-test was performed. Univariate and multivariate logistic regression analyses were performed to assess risk factors for a positive cryptococcal antigen test result. Risk factors with possible significance or those with biologic plausibility and known to be associated with cryptococcal disease were included in the model. Model building and selection was based on the purposeful selection of covariates strategy as previously described [12]. The sensitivity, specificity, negative and positive predictive for certain symptoms and combination of symptoms for a positive cryptococcal antigen test result were also determined. A p value <0.05 was considered statistically significant.

## Results

### Patients and antigen screening

A total of 369 HIV-infected patients in Addis Ababa were enrolled into the study (Table 1). The mean age of those enrolled was 36 years and 56% were female. The majority of patients were being prescribed ART (74%) and had been receiving ART for a mean duration of 34 months. The mean CD4 count was 123 cells/μl and 31% had a CD4 count less than 100 cells/μl. Over 70% of patients were enrolled from site 1 (ALERT). Only two patients had a prior history of cryptococcal disease (1%) in contrast to a high prevalence of prior pulmonary (25%) and extra pulmonary tuberculosis (9%). At least one clinical symptom was reported by 46% of patients with headache (28%) being the most common followed by fever (22%), night sweats (20%), and cough (18%). A total of 51 patients (14%) had a temperature ≥38.3°C recorded at the study visit.

**Table 1:** Baseline Characteristics of all 369 patients and comparison by cryptococcal antigen status.

Characteristic	Total (n = 369)	CRAG positive (n = 31)	CRAG negative (n = 338)	P*
Mean Age in years, (IQR)	36 (30–41)	40	36	0.02
Male (%)	163 (44)	19 (61)	144 (43)	0.05
Site 1 vs. 2 (%)	263 (72)	28 (90)	235 (70)	0.02
BMI <18.5 kg/m^2^ (%)	98 (27)	4 (13)	94 (28)	0.07
Mean months HIV diagnosis (IQR)	33 (5–59)	36	33	0.55
Taking ART (%)	271 (74)	26 (84)	245 (73)	0.19
Mean months on ART (IQR), n = 262	34 (8–58)	37	33	0.56
Mean CD4 count (IQR)	123 (82–167)	110	124	0.14
CD4 count status (%)				
<100	116 (31)	13 (42)	103 (31)	0.28*
100–150	113 (31)	10 (32)	103 (31)	
151–2000	140 (38)	8 (26)	132 (39)	
History of OIs (%)				
Cryptococcal disease	2 (1)	0	2 (1)	0.67
Pulmonary TB	91 (25)	11 (36)	80 (24)	0.14
Herpes Zoster	33 (9)	1 (3)	32 (10)	0.24
Current OIs (%)				
Pulmonary TB	24 (7)	2 (7)	22 (7)	0.99
Pneumonia or URI	9 (2)	0	9 (3)	0.36
Current Symptoms* (%)				
No symptoms	196 (54)	14 (48)	182 (56)	0.42
Fever	80 (22)	11 (36)	69 (21)	0.053
Headache	104 (28)	9 (29)	95 (28)	0.92
Neck Stiffness	11 (3)	2 (7)	9 (3)	0.24
Altered Mental Status	17 (5)	2 (7)	15 (4)	0.61
Photophobia	26 (7)	3 (10)	23 (7)	0.55
Nausea	44 (12)	6 (19)	38 (11)	0.18
Night sweats	72 (20)	7 (23)	65 (19)	0.65
Cough	65 (18)	6 (19)	59 (18)	0.80
Vomiting	31 (9)	4 (13)	27 (8)	0.35
Shortness of breath	29 (8)	0	29 (9)	0.09
Signs (%)				
Meningismus	4 (1)	1 (3)	3 (1)	0.23
Skin papules	15 (4)	0	15 (5)	0.23
Fever (≥38.3°C)	51 (14)	5 (16)	46 (14)	0.70
Cryptococcal antigen titer (%)				
1:8	-	5 (16)	-	-
1:16	-	8 (25)	-	-
1:32	-	9 (29)	-	-
1:64	-	3 (10)	-	-
1:128	-	3 (10)	-	-
1:1024	-	3 (10)	-	-

* Based on patient self-report; CRAG, serum cryptococcal antigen; BMI, body mass index; ART, antiretroviral therapy; OI, opportunistic infection; TB, tuberculosis; URI, upper respiratory infection acid; ^+^ANOVA Statistic.

**Figure 1:**
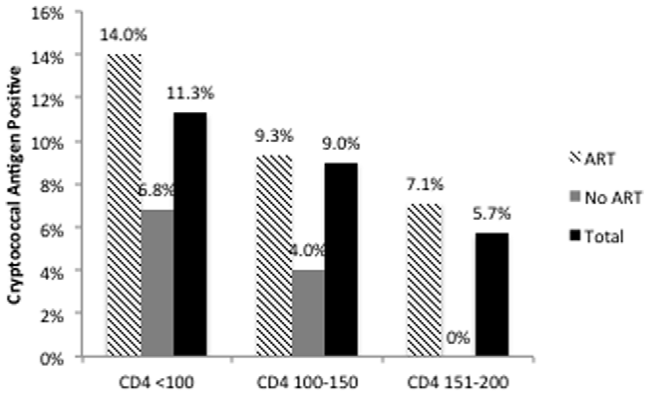
Percentage of HIV infected patients with cryptococcal antigenemia by CD4 count and antiretroviral use.

Among the 369 HIV-infected patients enrolled, 31 (8.4%) had a positive cryptococcal antigen test and all titers were 1∶8 or greater (Table 1). When stratified by CD4 count, 11% of patients with a CD4 <100 cells/μl had a positive cryptococcal antigen test as compared to 8.9% with CD4 between 100 to 150 cells/μl and 5.7% with CD4 >150 cell/μl (Figure 1). The large majority of patients with a positive cryptococcal antigen test (84%) were receiving ART. None of the patients with a positive cryptococcal antigen test had a known history of cryptococcal disease.

### Comparison of positive and negative antigen patients

Patients with a positive cryptococcal antigen test were significantly more likely to be older (40 vs. 36 years) and male (61% vs. 43%) as compared to those with a negative test. Patients with a positive cryptococcal antigen test were also much more likely to receive care at Site 1 [ALERT] (OR = 4.05, 95% CI 1.20–13.63). Patients with cryptococcal disease had a higher prevalence of fever than those with a negative test for cryptococcal disease but the difference did not reach statistical significance in univariate analysis (11/31 [36%] vs. 69/338 [21%], OR = 2.14, 95% CI 0.98–4.67, p = 0.06). In univariate analysis, there were no significant differences between those with and without a positive diagnostic test for cryptococcal disease for duration of HIV infection, receipt of ART, CD4 count status, and current or history of opportunistic infections and clinical symptoms and signs (Table 2).

**Table 2:** Univariate and multivariate analysis of risk factors for cryptococcal antigenemia among HIV infected patients in Addis Ababa, Ethiopia (n = 369).

Characteristic	Univariate Analysis		Multivariate Analysis	
	OR (95% CI)	P	OR (95% CI)	P
Age, per year	1.05 (1.01–1.09)	0.02	1.05 (1.002–1.09)	0.04
Male	2.12 (1.00–4.51)	0.05		
Site 1 vs. 2	4.05 (1.20–13.63)	0.02	5.49 (1.57–19.16)	0.01
BMI <18.5 kg/m^2^	0.39 (0.13–1.13)	0.08	0.32 (0.10–0.98)	0.046
Currently employed	1.33 (0.62–2.87)	0.46	-	-
On ART	1.91 (0.71–5.10)	0.20	2.60 (0.91–7.45)	0.08
CD4 count status				
<100	2.08 (0.83–5.21)	0.34	2.81 (1.06–7.47)	0.04
100–150	1.60 (0.61–4.20	0.11	1.81 (0.65–5.04)	0.26
151–200	1.00	-	1.00	-
Past OIs				
TB	1.16 (0.54–2.50)	0.71	-	-
Herpes Zoster	1.40 (0.40–4.95)	0.60	-	-
Current OIs (%)				
TB	0.71 (0.16–3.16)	0.65	-	-
Symptoms				
Fever	2.14 (0.98–4.67)	0.06	2.95 (1.26–6.87)	0.01
Headache	1.04 (0.46–2.35)	0.92	-	-
Photophobia	1.46 (0.41–5.18)	0.56	-	-
Signs				
Fever (≥38.3°C)	1.22 (0.45–3.33)	0.70	-	-
Meningismus	3.71 (0.37–36.79)	0.27	-	-

BMI, body mass index; ART, antiretroviral therapy; OI, opportunistic infection; TB, tuberculosis.

### Risk factors for a positive cryptococcal antigen test in multivariate analysis

In multivariate analysis, increasing age (aOR 1.05, 95% CI 1.002–1.09), clinic site 1 vs. 2 (aOR 5.49, 95% CI 1.57–19.16), fever (aOR 2.95, 95% CI 1.26–6.87) and a CD4 count <100 cells/μl as compared to a CD4 count >150 cells/μl (aOR 2.81, 95% CI 1.06–7.47) were all significantly associated with an increased risk of cryptococcal antigenemia (Table 2). Conversely, a BMI <18.5 kg/m^2^ was associated with a decreased risk of a cryptococcal antigenemia (aOR 0.32, 95% CI 0.10–0.98).

### Clinical symptoms and antigenemia

Results of an analysis of the performance of individual and combinations of four candidate symptoms in detecting cryptococcal antigenemia including negative and positive predictive value are shown in Table 3. There was poor sensitivity of any one symptom (10–36%) or combination of symptoms (9–43%) in detecting cryptococcal antigenemia. We also found less than optimal specificity of individual (72–93%) or combinations of symptoms (62–97%) for cryptococcal antigenemia.

**Table 3:** Predicting cryptococcal antigenemia based on the presence of individual or combination of clinical symptoms (n = 369).

Symptom(s)	Sensitivity (%)	Specificity (%)	PPV (%)	NPV (%)
Fever	36	80	14	93
Headache	29	72	9	92
Photophobia	10	93	12	92
Night Sweats	23	81	10	92
Fever, Headache, or Photophobia	42	62	10	92
Fever, Headache, and Photophobia	9	97	3	92

PPV, positive predictive value; NPV, negative predictive value.

## Discussion

Our study demonstrates a high prevalence of cryptococcal antigenemia among HIV-infected adults attending ART clinics in Addis Ababa, Ethiopia. Criteria for study entry was a CD4 count of ≤200 cells/μl; the overall prevalence of cryptococcal antigenemia was 8.4% and among patients with CD4 counts ≤100 cells/μl, the prevalence exceeded 11%. In multivariate analysis, increasing age, self reported fever, site of screening, and lower CD4 count (<100 cells/μl) were associated with an increased risk of having a positive serum cryptococcal antigen test.

These data are the first to describe the prevalence of cryptococcal antigenemia among HIV-infected patients in Ethiopia in the HAART era. The high prevalence of cryptococcal subclinical infection highlights the need for implementation of routine cryptococcal antigenemia screening in Ethiopia among HIV-infected persons. Current WHO guidelines recommend consideration of cryptococcal screening in high prevalence RLS among those with CD4<100 cells/μl and not on ART (followed by pre-emptive anti-fungal therapy if cryptococcal antigen positive) [1]. In contrast to prior studies done outside Ethiopia in Sub-Saharan Africa, [4], [8] we found a high prevalence of cryptococcal antigenemia (7.1%) among patients with CD4 counts between 100–200 cells/μl, which calls into question whether screening recommendations should be expanded to include patients with CD4 counts <200 cells/μl. We also found a previously unreported high prevalence of cryptococcal antigenemia among patients receiving ART and a significant effect of location on prevalence rates, results that may have implications for developing optimal cryptococcal screening prevention and treatment strategies. While our study found a CD4 count ≤100 cells/μl was significantly associated with cryptococcal antigenemia in multivariate analysis, the majority of positive patients (58%) in our study had a CD4 count between 100–200 cells/μl. Additionally, our prevalence rate of cryptococcal antigenemia (7.1%) in patients with a CD4 count >100 cells/μl was in contradiction to lower prevalence rates among patients with CD4 counts >100 cells/μl in South Africa (1.1%)^ 4^, Uganda (2.7%)^ 8^, and Thailand (3.6%)^ 7^. One reason we may have found a high prevalence of cryptococcal antigenemia among persons with a CD4 >100 is our inclusion of patients already on ART. These patients could have developed infection at lower CD4 counts prior to ART initiation and remained antigenemic as their CD4 count improved on ART. Another possibility is a high cryptococcal disease burden or other unique risk factors in Ethiopia. In contrast to prior studies that have evaluated cryptococcal antigenemia prior to ART initiation our study is the first study to our knowledge to describe cryptococcal antigen screening among HIV patients receiving ART. Of note, 26 (84%) of 31 patients with a positive cryptococcal antigen test were receiving ART and had been receiving for a mean duration of 37 months. Unfortunately, no data on HIV viral load was available on these patients and there was no way to assess if patients were responding appropriately to their ART regimen. While this may limit the generalization of our results, the current situation in SSA usually precludes healthcare providers from determining ART treatment failure thus making it difficult to base screening recommendations on ART response.

Our study adds to the mounting evidence for the public health importance of screening for cryptococcal antigenemia among HIV-infected adults in SSA and other RLS. Our overall prevalence of cryptococcal antigenemia (8.4%) is in line with results from Uganda (5–9%), South Africa (13%), and Kenya (6%) and reaffirms that in SSA high rates of cryptococcal disease are usually found when looked for. However, compared to tuberculosis (TB) prevalence, there are limited data on the magnitude of cryptococcal infection in SSA even though cryptococcal and TB disease have been estimated to be responsible for a similar mortality among HIV infected patients in SSA [2]. Our study is the first to estimate the prevalence of cryptococcal antigenemia in Ethiopia. Given Ethiopia has an estimated 1.1 million people living with HIV, the potential enormity of cryptococcal infection based on our results is significant and argues for rapid scale up of cryptococcal antigen screening among HIV-infected persons. Further large-scale studies of cryptococcal infection are currently underway in South Africa and are needed in other SSA and RLS countries to better understand the true extent of disease burden. Scale up of screening may be enhanced by the introduction of a FDA-approved cryptococcal antigen point of care test that has a high sensitivity and specificity [13], [14].

Our finding in multivariate logistic regression, after controlling for ART use and CD4 count, that patients at site 1 as compared to site 2 were more likely to have cryptococcal antigenemia (aOR 5.49, 95% CI 1.57–19.16) led us to question the effect of geography on cryptococcus exposure and disease prevalence. The ecological habitat of *Cryptococcus neoformans* has been found to include rotting wood and trees (including eucalyptus) and in soil contaminated by bird guano [15], [16]. Ethiopia has a large imported population of eucalyptus trees from Australia that could potentially serve as a source of infection. It is possible that patients attending site 1 as compared to site 2 had increased environmental exposure to *C. neoformans*; however, we did not collect detailed information on place of residence and it is also unclear if cryptococcal infection is from recent or past exposure to *C. neoformans*. Our intriguing finding generates more questions than answers and suggests that there may be hot spots for cryptococcal disease due to increased exposure or other yet unknown factors.

To assess the value of symptoms in detecting cryptococcal antigenemia we evaluated if the presence or absence of individual or combination of certain symptoms could predict or rule out cryptococcal antigenemia. This strategy has been used successfully in screening for TB in which the absence of current cough, fever, night sweats, and weight loss can reliably rule out TB among HIV infected patients in most RLS [17]. While persons who reported a fever were more likely to have cryptococcal antigenemia (aOR 2.95, 95% CI 1.26–6.87), 64% of positive patients reported no fever and 48% reported a lack of any symptoms. Additionally, we found non-optimal sensitivity, specificity, positive predictive value, and negative predictive value of any individual or combination of symptoms in detecting cryptococcal antigenemia. Our findings are similar to results from a prior study conducted in Uganda [9] and suggest that all adult HIV infected patients with low CD4 counts in high burdened settings should be tested for cryptococcal antigenemia regardless of symptoms.

The clinical relevance of cryptococcal screening has been established by studies that found a positive cryptococcal antigen test among ART naïve patients predicts the development of cryptococcal meningitis and is a strong risk factor for mortality [4], [5], [7]. Furthermore, Meya et al. demonstrated that pre-emptive therapy with fluconazole in asymptomatic cryptococcal antigen positive patients was a cost effective intervention. They found that 2–4 weeks of fluconazole (200–400 mg) along with ART initiation resulted in a 30-month survival of 71%. Additionally they calculated the cost to save one life using a test and treat strategy was $266 (with a cryptococcal antigenemia prevalence of 8.8%) [8]. The development of the cryptococcal antigen lateral flow assay (LFA) (Immy Inc, Norman, Oklahoma) has dramatically lowered Meya's estimated cost to save one life to $39.70 [3] and made the widespread roll out of cryptococcal antigen screening a more realistic goal. The cryptococcal antigen LFA is a FDA approved rapid point of care diagnostic test that is highly sensitive, affordable (∼$2), easy to perform and requires minimal infrastructure [14]. It has the potential to begin a new era of cryptococcal management in RLS. While cryptococcal screening and pre-emptive therapy appear to be cost effective interventions there remains many unanswered questions including the benefit of screening among ART experienced patients, how often to perform screening, and the optimal pre-emptive treatment regimen and duration.

Our study is subject to several limitations. These include lack of HIV viral load testing among patients enrolled in the study, which would have helped assess whether those on ART who had a positive diagnostic test for cryptococcal antigenemia were not controlled on their current ART regimen. Viral load testing is not routinely performed at the clinics where our study took place or in most HIV clinics in SSA. There was also an absence of lumbar punctures, which would help define the proportion of patients with cryptococcal meningitis among those with a positive cryptococcal antigen test, and no long-term clinical follow up of our patient cohort. The decision to perform lumbar puncture was left up to the treating physician and none were performed. Future studies will assess the rates of meningitis and clinical outcomes among those with a positive cryptococcal antigen. Finally, our study took place at only two HIV clinics in Addis Ababa; therefore further investigations are needed at HIV clinics in Ethiopia outside Addis Ababa to assess the prevalence of cryptococcal infection in areas throughout the country.

In conclusion, we found a previously unreported high prevalence of cryptococcal antigenemia among HIV-infected patients with CD counts ≤200 cells/μl, including those on ART in Ethiopia. Additional important findings include no utility of symptom screening in disease detection and differential risk of disease based on location. The results of the present study indicate the need to scale up cryptococcal antigen screening among HIV-infected persons in Ethiopia and suggest it may be beneficial to expand current cryptococcal screening recommendations. In addition, further exploration of the effect of location and environment on the risk of cryptococcal infection is warranted.
